# Antibacterial and Anticancer Activities of Fenugreek Seed Extract 

**DOI:** 10.31557/APJCP.2019.20.12.3771

**Published:** 2019

**Authors:** Lina A Naser Al-Timimi

**Affiliations:** *Department of Biology, College of Science, University of Basrah, Iraq. *

**Keywords:** Fenugreek seed, pathogenic bacteria, breast cancer, antibacterial effect

## Abstract

This work is about the utilization of fenugreek seed as an antibacterial and anticancer agents. The antibacterial activity of fenugreek seed extract on six pathological bacteria strains were specified through conventional biochemical tests using the Vitek2 automated system and diffusion agar method. The anticancer activities of fenugreek seed extract, on MCF-7 breast cancer cells, liver cancer HCAM cells and the non-cancerous Vero cell lines, were investigated using colorimetric MTT assay. Results showed that the highest activity of the extract of the seed was found on *Staphylococcus aureus* and *Pseudomonas aeruginosa* (22 mm and 17 mm diameter of inhibition zones respectively). The seed extract showed proliferative inhibition on MCF-7 cell line at a concentration of 400 µg/ml and 72 h of the incubation period. This was accompanied by insignificant apoptosis or necrosis. The seed extract showed no anticancer effect on liver and Vero cell lines. This work emphasizes that fenugreek seed extract is a potential source of antibacterial and anticancer agents.

## Introduction

Cancer is a serious health issue that concerns the entire world. Its treatment protocols depend on chemotherapy, radiotherapy and surgical intervention (Huang et al., 2014). Breast cancer stands out as one of the most threatening diseases to females around the world. In 2012, for example, 1.57 million cases were officially registered. Today, breast cancer is considered the second life threatening disease for women in the united states (Farshori et al., 2013; World Cancer Report, 2019). Chemotherapy is one of the most regularly adopted protocol in breast cancer treatment (Siegel et al., 2014). Treatment based on this protocol is usually accompanied with unfriendly symptoms, extending from sickness to bone marrow suppression to development of multidrug resistance (MDR) (Graidist et al., 2015; American Cancer Society, 2019). Therefore, discovering characteristic mixes from plants may provide an elective malignant growth treatment (Goldman et al., 2019).

In Iraq, breast cancer is becoming a major risk to women lives after cardiovascular diseases and leading to 23% of the overall death toll. Cancer has been threatening Iraqi women increasingly since 1986 (Alwan, 2010; Iraqi Cancer Board, 2015). In addition, the recurrence of this illness was observed in moderately aged women (45 – 50 years old) although the pinnacle age of exposure was accounted for to be in the range 50 – 55 years (Alwan, 2014; World Health Organization, 2017). Liver cancer is considered the third reason for disease mortality around the world (Farooq et al., 2013). According to recent study by WHO (2017) death with liver cancer in Iraq escalated to 693 representing 0.39% of the total death toll. It is worth mentioning that death toll in Iraq amounts to 4.25 per 100,000 placing Iraq in the 122nd positing worldwide (World Health Organization, 2017). 

The harmful nature of pathogenic bacteria may be controlled using antibiotics. Unfortunately, the misuse of antibiotics is leading to the speedy development of ineffectiveness of antibiotic strains to bacteria creating an alarming clinical status in the remedy of infections. Today, many infections occur because microorganisms defeat conventional therapy. In fact, bacteria have the genetic ability to develop resistance to many antibiotics (Fair and Tor, 2014; Landecker, 2015; Li and Webster, 2018).

Recently, there has been a surge interest in the antibacterial properties of the plants extract. It is progressively acceptable that these phytochemicals will be prescribed by the doctors as antibacterial medications (Bhalodia et al., 2011). Many investigations were carried out on the therapeutic applications of various plants species on different diseases such as fungal, viral, and bacterial contagion. Nowadays, approximately 33% of the world population depend on conventional/ therapeutic plants and their extract to meet the essential needs. At the same time, the world health organization (WHO) reported that 80% of individuals worldwide are accustomed to use manmade medications (Kumar and Reddy, 2012).

Recently, the study of the therapeutic effects of plants has increased due to their inclusive medicinal and economical properties and the successful utilization of some of these plants in treating human diseases. Some investigations have led to discovery of restorative properties of fenugreek seed. Fenugreek (Trigonella Foenum-gracium), also known in Arab countries as “Helba”, is a plant from the family of Leguminosae. It grows annually and is being planted in the Mediterranean countries and Asia. Fenugreek dried seed are known for their valuable antibacterial, anticancer and anti-inflammatory properties in India, Egypt and some European countries. These seed are also known to work as anti-oxidants having reviving properties. Fenugreek is rich with a wide variety of metabolites such as tannins, alkaloids, flavonoids, terpenoids and glycosides which are known to have antimicrobial properties (Khorshidian et al., 2016). 

In the present work it is aimed to investigate the antibacterial activity of fenugreek seed extract in general and the effect of ethanol fenugreek seed extract on breast cancer. This work is motivated with the fact that only few studies have been carried out on anticancer materials especially on MCF-7 cell line. 

## Materials and Methods

Fenugreek seed were procured from a herbal shop in Al-Ashar local market in Basrah city. The seed were initially washed, dried and then grinded using home mixer. The grinded seed were then mixed with ethanol and sterilized distilled water. For the biological purposes, 50 g of fenugreek seed powder was added to 500 ml of absolute ethanol. Another 50 g of the grinded seed were added to 500 ml of distilled water. The two mixtures were kept in a rotary shaker for 24 h and filtered with Whatman No.1 filter paper. A micro filter of 0.45 µm was then used in a rotary evaporator at 50°C for extra filtration. The extracted materials were stored at 4°C. 

Methanol: Dimethyl Sulfoxide (DMSO) in 1:1 V/V volume ratio was used to prepare different concentrations of 125, 250, 500 and 1,000 µg/ml crude plant seed ethanolic and aqueous extract. The final DMSO concentration did not exceed 0.1%.

The antibacterial activity of the extract were studied on six clinically isolated bacteria strains, these are *Staphylococcus aureus*, *Pseudomonas aeruginosa*, *Proteus mirabilis*,* Salmonella typhi*, *Escherichia coli*, and *Vibrio parahaemolyticus*. These strains were obtained from different sources (stool, wound infections, urine, skin lesions) of patients admitted to ‘Al-Shafaa hospital’ in Basrah. All the collected samples were processed upon receipt in laboratory and cultured in appropriate media (Manandhar et al., 2019).

In order to classify the isolated bacteria to Gram-negative and Gram-positive groups, staining, morphological identification of colonies under optical microscope and conventional biochemical tests using Vitek2 automated system, were performed . A bacterial suspension of each isolate was prepared and equalized to 0.5 McFarland standard and the solution was spread on the entire surface of Muller Hinton agar using a sterilized cotton bud (Barrow and Feltham, 2018). After drying, a 9 mm diameter pore was made in each plate by using cork-borer with duplicate and control plates. Approximately, 0.1 ml of each of the plant extract, with the concentrations mentioned above, were injected to fill the wells. All inoculated Petri dishes were incubated at 37°C overnight. The inhibition zone was then measured from the diameter of the clearing zone in millimeters. All the dishes were examined for the Minimum Inhibitory Concentration (MIC) that inhibits the bacterial growth after incubation (Bansode and Chavan, 2013).

All cell lines that used in this study were obtained from the Iraqi Center of Cancer and Medical Genetics Research (ICCMGR), Al-Mustansiryia University, including human breast cancer (MCF-7) and liver cancer (HCAM) in addition to the control serving non-cancerous Vero (green African monkey kidney) cell lines.

Different concentrations of ethanol fenugreek seed extract were determined by using (MTT) tetrazolium reduction assay (Aslantürk, 2017). For further biological inspections, the fenugreek seed extract was re-suspended in DMSO at 10.00 µg/ml stock solution. The concentration of the fenugreek seed extract, used to treat the cells, was in the range 400-1,000 µg/ ml. 20 ml of MTT solution was added to each well after 24, 48 and 72 hours of treatment. 

The Acridine Orange/Propidium Iodide (AO/PI) pigment was used for the phenotypically recognition of apoptosis, the cells passing were incited by concentrate. 

Morphological consideration includes nuclear fragmentation, membrane blabbing, cytosolic buildup. In addition, two hundred cells were tallied to each slide. The cells of untreated MCF-7 were considered a control. The inhibition rate (IR) of cell growth was calculated by counting the percentage of proliferation rate (PR). PR = B/A where A refers to optical thickness of the untreated wells and B refers to the optical thickness of the treated wells. IR=100-PR (Gao et al., 2005).

In order to rate the apoptosis, the existence of inter-nucleosome DNA cleavage was visualized against a DNA ladder in agarose gel electrophoresis using biochemical markers. Breast cancer cells line MCF-7 and HCAM were cultured in two 25 cm cell culture flasks for 24 h before being treated with fenugreek extract. All the cells were processed with fenugreek extract overnight. Then, MCF-7 and HCAM were collected, washed with PBS and filtered using a DNA filtration, DNeasy and Tissue kits. By using electrophoresis, the DNA was resolved at 80–100 V through 1.8 % of agarose gel. The gel was recolored using ethidium bromide and imaged through ultra-violate trans illuminator.

## Results

*Antibacterial activity*

**Figure 1:**
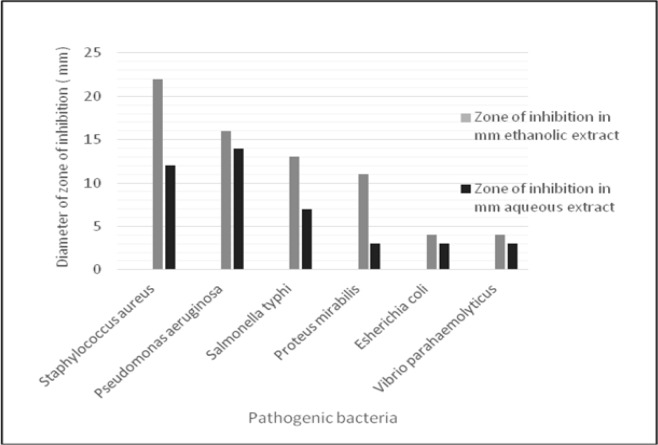
Antimicrobial Activity of Different Concentrations of 125, 250, 500 And 1,000 µG/Ml Ethanol and Aqueous Fenugreek Seed Extract on Bacterial after Incubation For 24h

**Figure 2:**
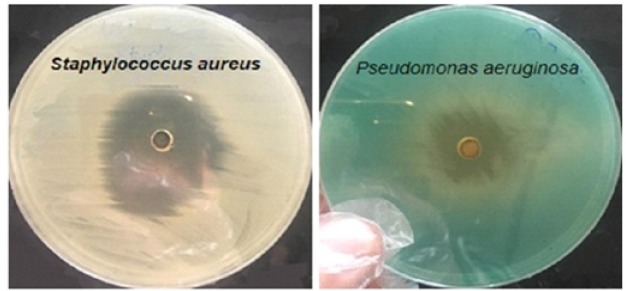
Antimicrobial Activity of Ethanol Fenugreek Seed Extract on *Staphylococcus aureus* and *Pseudomonas aeruginosa* in Muller-Hinton Agar

The antibacterial activity of fenugreek seed extract, evaluated in terms of inhibition zone, was tested on six pathogenic bacteria. The MIC results are shown in Figures 1, 2 and Table 1. Results showed that ethanol extract has prominent effect on Gram positive S. aureus and Gram negative P. aeruginosa. At the same time this extract showed moderate activity on the remaining types of the bacteria. It is worth noting that none of the prepared concentrations of ethanol extract showed positive activity on Vibrio parahaemolyticus and* E. coli*. On the other hand, the aqueous extract showed low to moderate activities on the bacteria except on *Staphylococcus aureus *and *E. coli*. The concentrations used in MIC for ethanol extract were in the range 50-500 µg/ml. Most of the bacteria were inhibited at MIC of 50 µg/ml. These results are significantly important due to the little amount of the fenugreek seed extract needed to inhibit the growth of the bacteria. On the other hand, none of the MIC values were exhibited by Vibrio parahaemolyticus in all the extract.

*Cytotoxicity assessment*

A cytotoxicity assessment of ethanol fenugreek seed extract using MTT technique was carried out. In this part of the work, the anticancer activity of the fenugreek seed extract was measured on MCF-7 and HCAM; the normal Vero cell line was used as a control. The tested concentrations for each cell line were 400, 600, 800 and 1,000 µg/ml. The incubation period was 72 h. The MTT assay revealed that ethanol fenugreek extract had an inhibitory action on MCF-7 cell line which amounts to more than half of the inhibition of the MCF-7 cell. Proliferation stopped when cell line concentration was equal to 400 µg/ml after 72 h of incubation. On the other hand, no cytotoxic effect of the extract was observed on liver cancer or Vero cell lines regardless of the concentration in action. It is worth mentioning that apoptosis was determined by using AO/PI recoloring and DNA fragmentation test. 

In this study, breast cancer cells treated with fenugreek seed extract showed that a perfect DNA ladder style was not clear in a time and concentration-dependent manner. 

This may indicate that the cells were inhibited and no apoptosis or necrosis occurred. Figure 4 shows genomic DNA isolated from MCF-7 cell line after being treated with fenugreek seed extract. After staining using ethidium bromide in agarose gel, no evidence of DNA fragmentation was observed. With respect to the AO/PI coloring, the present study showed no death of MCF-7 cell line after being treated with ethanol fenugreek seed extract. This emphasizes that MCF-7 cells inhibition occurs without any apoptosis or necrosis (see Figure 5). 

**Figure 3:**
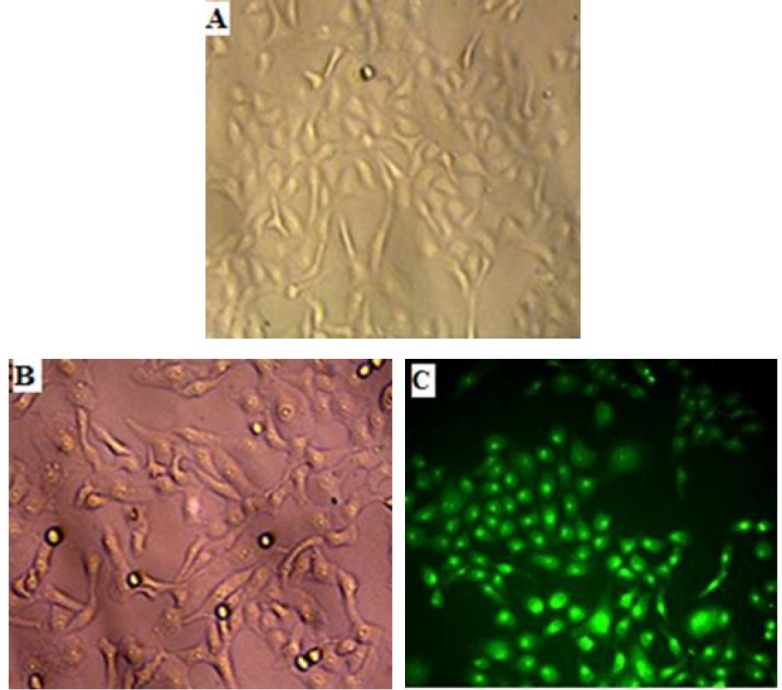
Vero cell line. A, Control under light microscope (magnified at 10x); B, After treated with different concentrations of 400, 600, 800 and 1,000 µg/ml ethanol fenugreek seed extract after 72 h under light microscope (magnified at 10x); C, After treated with different concentrations of 400, 600, 800 and 1,000 µg/ml ethanol fenugreek seed extract after 72 h pigmented with the AO/PI. Notice there is no effect on the cell after treated. Viewed under 690 wavelength of the fluorescent microscope light microscope (magnified at 10x)

**Figure 4:**
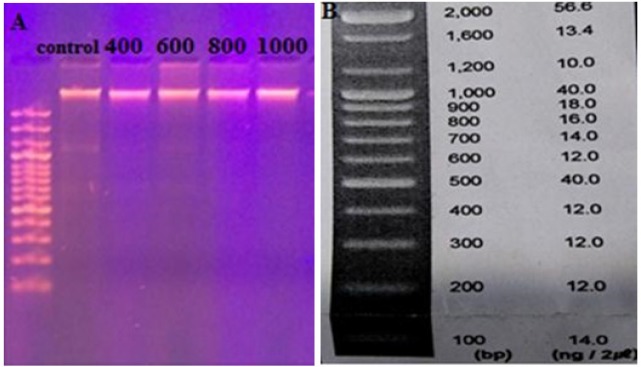
A, Genomic DNA isolated from MCF-7 cell line after being treated with different concentrations of 400, 600,800 and 1000 µg/ml ethanol fenugreek seed extract after 72 h; B, The ladder used its range from 100-2,000 (bp)

**Figure 5:**
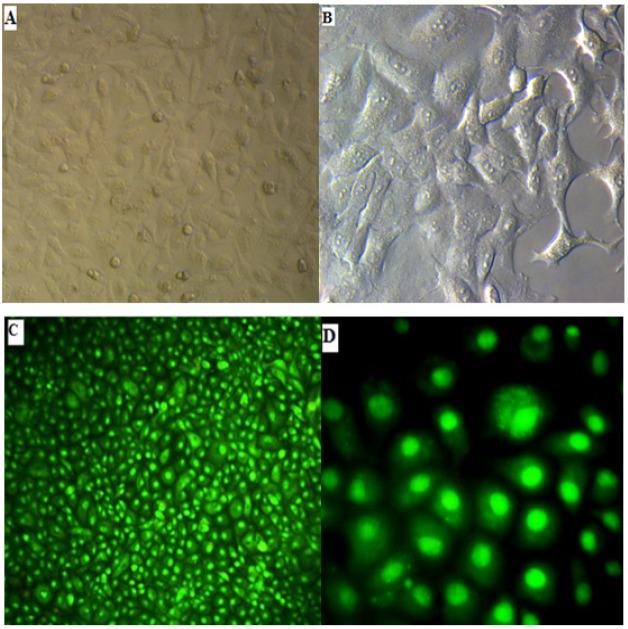
Human breast cancer cells (MCF7). A and B, Control viewed under light microscope the magnification force for A is 10x and 40x for B; C and D, MCF-7 cells treated with ethanol fenugreek seed extract at a concentration of 400 µg/ml and 72 h pigmented with the AO/PI, notice their color green only. Shown by the 690 wavelength of the fluorescent microscope, the magnification force is 10x for C and 40x for D

**Table 1:** The Minimum Inhibitory Concentration Rate (MIC) Of Different Concentrations Of 125, 250, 500 And 1,000 µG/Ml Of Fenugreek Seed Ethanolic Extract Against Bacterial Test Organisms

Bacteria	MIC of ethanol extract (µg/ml)			
	500	250	100	50
*Staphylococcus aureus*	+	-	+	-
*Pseudomonas aeruginosa*	+	-	+	-
*Salmonella typhi*	+	-	-	-
*Proteus mirabilis*	+	-	-	-
*Escherichia coli*	+	-	-	-
*Vibrio parahaemolyticus*	-	-	-	-

## Discussion

There is an urgent need to find new antibacterial medications with novel characteristics to tackle the rise of new infectious diseases and due to the misuse of conventional antibiotics. There is, therefore, interesting progress in extracting certain chemicals from different plants. Plants are known to provide wide spectrum of chemical compounds having various biological activities. Antibiotics of medicinal plants have contributed to the elimination of many diseases caused by pathogenic bacteria. The importance of these plants can be demonstrated by the fact that these may be designed as target-oriented materials unlike chemical antibiotics playing the role of plant versus drug-resistant bacterial diseases (Amenu, 2014).

Fenugreek is a herbal plant. Its fresh seed, twigs, roots, and leaves can be used directly and after being dried as flavoring, supplements and spices. Its medical advantages have been reported in many studies. Infections by *Staphylococcus aureus *and *Pseudomonas aeruginosa* has become a serious issue in sick and immune-compromised patients. For this reason, the antibacterial effect of fenugreek ethanol extract on these strains receives significant interest. The serious issue prompting high mortality lies in the presence of drug-resistant strains (Amenu, 2014; Bassetti et al., 2018). In the present investigation both Gram-positive and Gram-negative bacterial strains exhibited similar response after exposure to fenugreek seed extract. This agrees with the work of Bassetti et al. (Alwan et al., 2017). On the other hand, ethanol extract exhibited higher activity on most of the bacterial strains except* E. coli* compared with the aqueous extract. This is agreeing with (Alwan et al., 2017) but disagree with (Salah et al., 2010; Sharma et al., 2017) where they recorded both extraction of ethanol and aqueous didn’t exhibit any effect on bacterial species.

Breast cancer is one of the most life-threatening disorder being suffered by Iraqi young women. In 1986, breast cancer was considered the top most serious malignancy threatening the Iraqi population (International Agency for Research on Cancer, 2013). In the present study, fenugreek seed extract showed cytotoxic activity which inhibits the MCF-7 cell development. Such effect was not observed on liver cancer cell lines. This emphasizes that the effect of fenugreek seed extract is cell type-dependent. These results comply with other results reported in the literature (Amin et al., 2005; Kyung et al., 2006). These antithetic results may be attributed to the specific activity of fenugreek on transformed and untransformed cells. Nevertheless, our results emphasize that fenugreek plant can be used in breast cancer treatment. Kyung et al., (2011) have reported similar findings in their work. It is worth mentioning that breast cancer MCF-7 cells can resist chemotherapy as they contain the CASP-3 gene which prompts an acquired insufficiency of caspase-3. Caspase-3 usually functions via death indexes and cleaves a variety of serious cellular proteins. DNA cleavage and a portion of the particular morphological features are in charge of apoptotic cells such as budding and shrinkage (Shapiro et al., 2001; Sharief and Gani, 2004). 

The impact of cytotoxic compounds demonstrates the dissemination of cell populations during the cells life span. Due to the significance of cell life span in cancer progression, few researchers have attempted to describe the cell cycle capture limit to the plant extract in addition to the importance of isolated compounds (Bassermann et al., 2014; Ruijtenberg and Heuvel, 2016). In the present work, no DNA fragmentation occurs as reported by other researchers (Shapiro et al., 2001; Sharief and Gani, 2004). When MCF-7 cell lines experience apoptosis with no evidence of DNA fragmentation this might be attributed to the absence of caspase-3. In the present work, normal Vero cells were used for the differentiation between normal and cancerous cell patterns. Furthermore, ethanol fenugreek seed extract showed no cytotoxic activity.

In conclusion, fenugreek seed are potential sources to new antibacterial compounds as emphasized from the antibacterial activity of their aqueous and ethanol extract on many pathogenic bacterial strains. Furthermore, fenugreek seed extract has shown anticancer activity through inhibition of more than half of the human breast cancer MCF-7 cell lines. These seed extract inhibited cancer cell’s proliferation with no apoptosis or necrosis observed. Further studies are still required to understand the mechanism (Shapiro et al., 2001; Sharief and Gani, 2004). of cells inhibition. This positive results on MCF-7 cell lines suggest that fenugreek seed extract is a potential anticancer agent that should be exploited widely for breast cancer treatment. 

## Conﬂict of interest

All contributing authors declare no conﬂicts of interest.
